# The performance of lipid profiles and ratios as a predictor of arterial stiffness measured by brachial-ankle pulse wave velocity in type 2 diabetic patients

**DOI:** 10.12688/f1000research.128627.1

**Published:** 2022-12-23

**Authors:** Soebagijo Adi Soelistijo, Robert Dwitama Adiwinoto, Agung Pranoto, Deasy Ardiany

**Affiliations:** 1 Division of Endocrinology, Diabetes, and Metabolism, Department of Internal Medicine, Airlangga University Faculty of Medicine, Dr. Soetomo Academic Teaching Hospital, Surabaya, 60132, Indonesia; 2 Department of Internal Medicine, Airlangga University Faculty of Medicine, Dr. Soetomo Academic Teaching Hospital, Surabaya, 60132, Indonesia

**Keywords:** dyslipidemia, arterial stiffness, cardiovascular disease, risk, type 2 diabetes mellitus

## Abstract

**Background:** Early identification of arterial stiffness in Type 2 diabetes mellitus (T2DM) patients before the manifestation of atherosclerosis would be clinically beneficial. Our study aimed to explore the correlation of lipid profiles and ratios with arterial stiffness, and construct a predictive model for arterial stiffness in T2DM patients using those parameters.

**Methods:** One hundred and eighty-four adult T2DM patients in the diabetes outpatient clinic at the Dr. Soetomo general academic hospital were enrolled in this cross-sectional study in 2015 and 2019. Sociodemographic, glycosylated hemoglobin (HbA1c), lipid profiles, and brachial-ankle pulse wave velocity (ba-PWV) data were collected from all subjects. The subjects were divided into a group with arterial stiffness (ba-PWV > 18 m/sec) and without arterial stiffness (ba-PWV ≤ 18 m/sec). A correlation test was used to evaluate the association, and receiver operator characteristics (ROC) curves analysis were used to determine the cut-off value, sensitivity, and specificity. The risk analysis model was calculated using bivariate logistic regression analysis.

**Results:** The group with arterial stiffness had higher lipid profiles: total cholesterol (TC), triglyceride (TG), high-density lipoprotein cholesterol (HDL-C), low-density lipoprotein cholesterol (LDL-C), and lipid ratios. A significant positive correlation was found between TC, TG, LDL-C, and all lipid ratios with ba-PWV. A negative correlation was found between HDL-C and ba-PWV. All lipid ratio parameters can be used as predictors of arterial stiffness, especially non-HDL-C with cut-off value: 150 mg/dL (sensitivity 96.8% and specificity 52.9%) and TG/HDL-C ratio with cut-off value: 4.51 (sensitivity 81.0% and specificity 74.2%). Elevated TG/HDL-C ratio and non-HDL-C displayed higher risk (OR: 12.293 and 16.312; *p* < 0.05) of having arterial stiffness compared to other lipid ratios.

**Conclusions:** Lipid profiles and lipid ratios, especially TG/HDL-C ratio and non-HDL-C, are potential biochemical markers for arterial stiffness in T2DM patients.

## Introduction

Type 2 diabetes mellitus (T2DM) is a major health concern and prevalence is increasing worldwide. More than 460 million people are already affected, or 9.3% of the global population, and there are projected to be around 578 million cases by 2030. Indonesia is among the top ten countries with the most prevalent occurrence of diabetes, estimated to be around 10.7 million cases in 2019 and projected to increase to 13.7 million in 2030 and to 16.6 million in 2045. ^1^^,^^2^ The consequence of the growing rates of T2DM is the increase in diabetes-related complications, especially macrovascular and microvascular. The prevalence of macrovascular and microvascular complications was estimated to be 27.2% and 53.5%, respectively. ^3^ However, cardiovascular disease (CVD) remains the leading cause of morbidity and mortality in T2DM patients. ^4^ The economic impact of T2DM regarding the medical care costs and the indirect costs due to decreased productivity associated with diabetes-related morbidity is also significant. ^5^

Coronary artery disease, peripheral artery disease (PAD), heart failure, and cerebral infarction are the typical manifestations of atherosclerotic CVD (ASCVD) in T2DM. Abnormal lipid metabolism (dyslipidemia) is the main pathogenesis for the development of ASCVD; meanwhile dyslipidemia is common in T2DM patients, affecting 60–70% of T2DM. ^6^ The association between cholesterol and the cardiovascular outcome is well established. Recently, many risk factors for CVD have been published in international studies, implying more complex lipoprotein disorders in atherosclerosis development. Lipid ratios (TC/HDL-C, TG/HDL-C, and LDL-C/HDL-C) application, in terms of cardiovascular risk stratification and lipid-lowering therapy efficacy assessment, are promising options. Besides the advantage of being practical in clinical settings, changes in these ratios have indicated better measures of the reduction in cardiovascular risk compared with the conventional lipid parameters. ^7^

Arterial stiffening depicts degenerative changes of the extracellular matrix (ECM) in the arterial media layer, marked with collagen deposition, cross-linking, and elastin fatigue fracture. From a pathological perspective, arterial stiffening is different from atherosclerosis. However, both processes commonly occur in the same vascular area, are part of the vascular aging process, and share the same risk factors. Moreover, several clinical studies have reported a significant association between arterial stiffness and the degree of atherosclerosis and also with the risk of cardiovascular event incidents. ^8^ Early recognition of arterial stiffness in the high-risk population, such as T2DM, before the manifestation of clinical ASCVD, is substantially beneficial. ^9^ Carotid-femoral pulse wave velocity (cf-PWV) represents primarily the aortic stiffness and is considered the gold standard for arterial stiffness measurement. ^10^ Nowadays, ba-PWV has been widely used in clinical settings and it has strong positive association with cf-PWV. Because ba-PWV is easier to obtain and more convenient for patients, it could serve as an alternative to cf-PWV measurement. ^11^^,^^12^

Serum HDL-C, in the middle-aged and elderly population, has been associated with protection against arterial stiffness. Previous studies have also investigated the degree of association between a single atherogenic lipid parameter or ratio (the LDL-C, TG/HDL-C, and non HDL-C) with arterial stiffness. However, each of those studies claimed some lipid parameters or ratios superior to one another. ^9^ Furthermore, the data regarding the use of those lipid ratios as a predictive biochemical marker for arterial stiffness in T2DM are lacking, especially in the Indonesian population.

## Objectives

Therefore, this study aimed to explore the correlation of lipid profiles and ratios with arterial stiffness, construct a predictive model for arterial stiffness in T2DM patients, and ultimately analyze the performance (sensitivity and specificity) of the lipid ratios to detect arterial stiffness in T2DM patients.

## Methods

### Ethical considerations

This study was approved by the ethical committee of Dr. Soetomo General Academic Hospital, Surabaya, Indonesia: 316/Panke.KKE/V/2015 (May 29 ^th^, 2015) and 1311/KEPK/V/2019 (July 20 ^th^, 2019). After receiving an explanation from the researchers about the research procedure, all eligible subjects gave written informed consent.

### Study design and setting

This was a cross-sectional design study conducted in the Endocrinology and Diabetes outpatient clinic at the Dr. Soetomo General Academic Hospital in Surabaya, Indonesia, during February–May 2016 and September–November 2019.

### Participants

The sample size of this study was calculated using the following formula to determine the minimum sample needed.

$$n={\left[\frac{\left(\mathrm{Z\alpha}+\mathrm{Z\beta}\right)}{0.5\ln\frac{1+r}{1-r}}\right]}^{2}+3$$

Significance = 95%; power = 80%; correlation coefficient = 0.217 from previous study. ^9^ Therefore, the minimum number of subjects for this study was 165. The subjects were recruited consecutively according to the inclusion and exclusion criteria. The inclusion criteria were adult patients (over 18 years of age) who had been diagnosed with T2DM. The exclusion criteria were current smoker, end-stage, or chronic kidney disease (CKD), subjects with active infection, known PAD, and subjects with a recent history of cerebral infarction (within 30 days).

### Study variables

The dependent variable was arterial stiffness measured by ba-PWV. The independent variables were lipid profiles (total cholesterol, triglyceride, HDL-C, LDL-C, and non-HDL-C) and lipid ratios (TC/HDL-C, TG/HDL-C, and LDL-C/HDL-C).

### Data collection

All participants were subjected to complete history, physical examination, and ba-PWV measurement. Lipid profiles and HbA1c data were obtained from the recent blood biochemistry examination in the last three months from the medical record, should the data be available. Otherwise, a venous blood sample was collected and sent to the laboratory of Dr. Soetomo General Academic Hospital in Surabaya, Indonesia for biochemical analysis. The TC/HDL-C is the ratio of the value of TC levels divided with HDL-C levels. The TG/HDL-C ratio is the result of the value of TG levels divided with HDL-C levels. The LDL-C/HDL-C ratio is the result of the value of LDL-C levels divided with HDL-C levels. The Non-HDL cholesterol value was calculated from HDL-C subtracted with TC levels. ^13^

### Brachial-ankle pulse wave velocity measurement

Pulse wave velocity is calculated from the ratio of the distance between two measurement points divided by the time required for the pressure wave to travel the distance. The travel distance was defined as the measurement from the center of the brachial cuff to the center of the ankle cuff. The pulse transit time (PTT) was obtained from two peak points of the diastolic pulse wave. ^8^^,^^12^ Brachial-ankle pulse wave velocity measurement was carried out using Va-Sera VS-1000 (Fukuda Denshi, Tokyo, Japan). The cut-off value of ba-PWV to define arterial stiffness is 18 m/sec. ^14^

### Statistical analysis

Data were analyzed using SPSS version 22.0 for Windows (IBM Corporation, New York, USA). Descriptive statistics for data that were normally distributed were expressed using mean and standard deviation, otherwise median and minimum maximum were used. An independent t-test or Mann–Whitney U-test was used to detect differences in lipid profile and lipid ratio values between groups with and without arterial stiffness. The correlation between HbA1c levels, lipid profiles, and lipid ratios with arterial stiffness was evaluated using Pearson or Spearman correlation test. Analysis of receiver operating characteristic curves (ROC) were used to determine the cut-off value, sensitivity, and specificity for each lipid ratio for arterial stiffness. Finally, bivariate logistic regression analysis was used to estimate risk prediction of lipid ratios against arterial stiffness in T2DM. Statistical significance was considered at *p* value < 0.05 and 95% confidence interval (CI).

## Results

### Subject characteristics

A total of 184 T2DM patients matched the inclusion criteria. ^15^ There were no significant differences in the sociodemographic (age and sex) and clinical (hypertension and obesity) characteristics between groups with and without arterial stiffness ( *p* > 0.05). The HbA1c levels in the group with arterial stiffness appeared to be higher than the group without arterial stiffness and the difference was significant ( *p* = 0.038). The lipid profile result was significantly higher in the group with arterial stiffness ( *p* < 0.05) except for the LDL-C levels ( *p* = 0.261). The HDL-C levels were significantly lower in the group with arterial stiffness compared to the group without arterial stiffness ( *p* < 0.05). Lipid ratio findings were also significantly higher in the group with arterial stiffness. The comparison is presented in Table 1.

**Table 1.** Comparison of demographic characteristics, obesity, glycemic control, lipid profile, and lipid ratios between groups with and without arterial stiffness.

Characteristics	Arterial stiffness present (baPWV > 18 m/s) n = 31	Arterial stiffness absent (baPWV ≤ 18 m/s) n = 153	*p*
Age (years), median (min–max)	63 (40–71)	57 (18–77)	0.186
Sex (n, %)			0.938
Male	13 (17.1%)	63 (82.9%)	
Female	18 (16.67%)	90 (83.33%)	
Hypertension (n, %)			0.558
Yes	19 (18.27%)	85 (81.73%)	
No	12 (15%)	68 (85%)	
Obese (n, %)			0.483
Yes	7 (13.72%)	44 (86.28%)	
No	24 (18.04%)	109 (81.96%)	
HbA1c (%), median (min–max)	8.4 (6.2–12.5)	7.6 (5.2–12.1)	0.038 *
Total cholesterol (mg/dL), median (min–max)	212 (160–300)	197 (100–350)	0.000 *
Triglyceride (mg/dL), median (min–max)	190 (91–380)	141 (38–412)	0.000 *
HDL-C (mg/dL), median (min–max)	42 (28–82)	47 (29–157)	0.005 *
LDL-C (mg/dL) (mean±SD)	141.29±31.04	132.59±40.64	0.261
Lipid ratios			
TC/HDL-C (mean±SD)	5.41±1.3	4.14±1.19	0.000 *
TG/HDL-C (mean±SD)	4.8±1.58	3.26±1.69	0.000 *
LDL-C/HDL-C (mean±SD)	3.41±1.02	2.84±1.1	0.008 *
Non HDL-C (mean±SD)	181.29±29.76	146.77±42.13	0.000 *

baPWV: brachial-ankle pulse wave velocity, HbA1c: glycosylated hemoglobin, HDL-C: high-density lipoprotein, LDL-C: low-density lipoprotein, TC: total cholesterol; TG: triglyceride.

* Significant ( *p* < 0.05).

### Correlation of lipid profiles and lipid ratios with ba-PWV

The Spearman correlation test was used to evaluate the correlation between HbA1c, lipid profiles and lipid ratios with ba-PWV values since the ba-PWV values were not normally distributed. We did not find a significant correlation between HbA1c and ba-PWV ( *p* > 0.05). The lipid profiles and lipid ratios were significantly correlated with ba-PWV ( *p* < 0.05) with various degrees of correlation. A negative correlation was found between HDL-C levels and ba-PWV values (ρ = -0.221) implying an inverse correlation. Lipid profiles (TC, TG, HDL-C, and LDL-C) had a weak correlation with ba-PWV when used alone. However, an increase in the correlation coefficient was observed when used as lipid ratios (TC/HDL-C, TG/HDL-C, LDL-C/HDL-C, and non HDL-C). The TC/HDL-C ratio demonstrated a moderate correlation (ρ = 0.419) among other lipid ratios. The correlation is described in Table 2.

**Table 2.** Correlation of glycemic control, lipid profile, and lipid ratios with brachial-ankle pulse wave velocity.

Parameter	ρ	*p*
HbA1c (%)	0.048	0.515
Total cholesterol (mg/dL)	0.292	0.000 *
Triglyceride (mg/dL)	0.239	0.001 *
HDL-C (mg/dL)	-0.221	0.003 *
LDL-C (mg/dL)	0.166	0.024 *
TC/HDL-C	0.419	0.000 *
TG/HDL-C	0.326	0.000 *
LDL-C/HDL-C	0.281	0.000 *
Non HDL-C	0.371	0.000 *

HbA1c: glycosylated hemoglobin, HDL-C: high-density lipoprotein, LDL-C: low-density lipoprotein, TC: total cholesterol, TG: triglyceride. Spearman correlation test was used.

* Significant ( *p* < 0.05).

### Model of lipid ratios as predictive markers for arterial stiffness in type 2 diabetes mellitus

**Figure 1.**
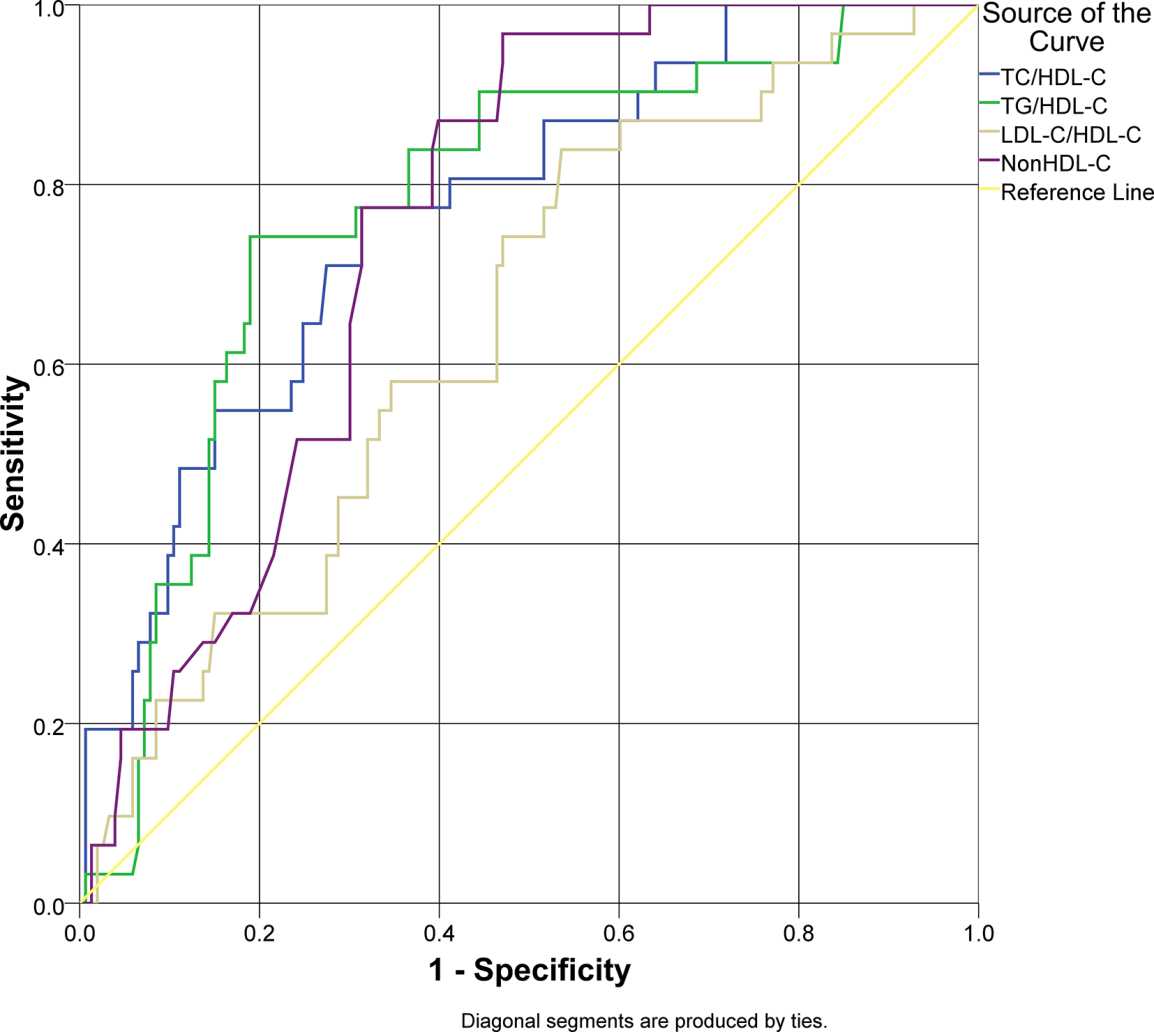
Receiver operator characteristics curve analysis of lipid ratios as a predictor of arterial stiffness in type 2 diabetes mellitus.

An ROC analysis was applied for lipid ratios to arterial stiffness as the outcome of this study ( Figure 1). Overall lipid ratios TC/HDL-C, TG/HDL-C, and non-HDL-C had a good performance to be used as predictive models (AUC > 0.7), except for LDL-C/HDL-C. Non-HDL-C had a sensitivity of 96.8% for arterial stiffness; however, the specificity was 52.9% with cut-off value 150 mg/dL. TG/HDL-C ratio had a specificity of 81.0% and sensitivity of 74.2% with cut-off value 4.51. The characteristics of the lipid ratios are presented in Table 3.

**Table 3.** Characteristics of lipid ratios using the optimal cut-off value to detect the presence of arterial stiffness in type 2 diabetes mellitus.

Parameter	AUC	95% CI	Cut-off value	Sensitivity	Specificity	*p*
TC/HDL-C	0.769	0.682–0.856	4.63	77.4%	68.6%	0.000 *
TG/HDL-C	0.776	0.688–0.863	4.51	74.2%	81.0%	0.000 *
LDL-C/HDL-C	0.644	0.544–0.744	2.71	83.9%	46.4%	0.012 *
Non HDL-C	0.753	0.679–0.827	150	96.8	52.9%	0.000 *

AUC: area under the curve, CI: confidence interval, HDL-C: high-density lipoprotein, LDL-C: low-density lipoprotein, TC: total cholesterol, TG: triglyceride.

* Significant ( *p* < 0.05).

### Risk analysis model of lipid ratios as predictor for arterial stiffness in type 2 diabetes mellitus

The cut-off value of each lipid ratio derived from the ROC curve was used to determine the presence of arterial stiffness. Values above the cut-off point were classified as elevated, while values below or equal to the cut-off point were classified as normal. All lipid ratios demonstrated significant association as a risk factor for arterial stiffness in T2DM patients through bivariate logistic regression analysis (OR > 1; *p* < 0.05). Elevated TG/HDL-C ratio and non-HDL-C displayed higher risk (OR 12.293 and 16.312) of having arterial stiffness compared to elevated TC/HDL-C or LDL-C/HDL-C ratio (OR 5.347 and 4.502). The risk analysis model of the lipid ratios is presented in Table 4.

**Table 4.** Binary logistic regression analysis model of lipid ratios for having arterial stiffness in type 2 diabetes mellitus.

Parameter	β	OR	95% CI	*p*
TC/HDL-C	1.677	5.347	2.291–12.478	0.000 *
TG/HDL-C	2.509	12.293	4.996–30.247	0.000 *
LDL-C/HDL-C	1.505	4.502	1.642–12.343	0.003 *
Non HDL-C	2.792	16.312	3.760–70.780	0.000 *

OR: odds ratio, CI: confidence interval, HDL-C: high-density lipoprotein, LDL-C: low-density lipoprotein, TC: total cholesterol, TG: triglyceride.

* Significant ( *p* < 0.05).

## Discussion

To our knowledge, our present study is the first to evaluate not only the correlation of lipid ratios with arterial stiffness in T2DM patients but also define the cut-off value of lipid ratios which might contribute new insight in managing dyslipidemia and CVD risk in T2DM. The main findings of our study were: 1) the lipid ratios were correlated with arterial stiffness; 2) the lipid ratios, especially TG/HDL-C ratio and non-HDL-C, had good sensitivity to detect arterial stiffness; 3) elevated TG/HDL-C ratio and non HDL-C levels increased the risk of having arterial stiffness in T2DM. These findings indicate that lipid ratios are independently associated with the risk of arterial stiffness in the T2DM population.

Atherosclerosis is the main pathogenesis of CVD; however, early atherosclerosis is asymptomatic, therefore the majority goes undetected in the early stages. Arterial stiffness is involved in these early stages of atherosclerosis. Even though the relationship between arterial stiffness and atherosclerosis is still unclear, the interaction between the two may involve several complex hemodynamic, mechanical, metabolic, and enzymatic pathways. The increase in arterial stiffness results in increased blood pressure which promotes arterial remodeling leading to atherosclerosis. ^16^ The increase in intra-luminal pressure also promotes atheroma formation and deposition of excessive collagen in arterial walls. ^8^ These mechanisms are even more pronounced in T2DM in which endothelial dysfunction, a pivotal event in the initiation of atherosclerosis, is accelerated by the presence of hyperglycemia and advanced glycation end products (AGEs). ^17^ Moreover, T2DM is characterized by abnormal metabolism of lipoprotein which contributes to the pathomechanism of atherosclerosis. Therefore, the detection of arterial stiffness in T2DM before the development of clinical ASCVD is crucial.

Dyslipidemia and DM commonly occur together, affecting 60–70% of people with T2DM. The pathophysiology of dyslipidemia in T2DM is complex, involving a different number of abnormalities. The key abnormality is the increased production of very low-density lipoprotein (VLDL) by the liver, which is commonly found as elevated TG levels. Elevated TG-rich lipoproteins will affect other lipoproteins and result in lower Apo A-I and HDL-C levels. Increased LDL-C association with CVD is well established in T2DM patients, as in the non-diabetic population. However, a significantly reduced LDL-C still carries residual CVD risk in T2DM. ^18^ Therefore, a new CVD risk predictor is needed, and lipoprotein ratios can provide a better picture of the complex metabolic and clinical interactions between conventional lipid profile parameters. This is consistent with our findings in which lipid ratios showed a higher correlation with ba-PWV compared to conventional lipid profile parameters alone.

In our study, a significant correlation between TG/HDL-C ratio and arterial stiffness were observed (ρ = 0.326). Moreover, the TG/HDL-C ratio of > 4.51 predicts arterial stiffness with a sensitivity of 74.2% and specificity of 81.0%. An elevated TG/HDL-C ratio also increased the risk for arterial stiffness (OR: 12.293; 95% CI: 4.996–30.247). These findings are consistent with the study conducted by Chen & Dai ^19^ and Zhao *et al*. ^9^ which found that TG/HDL-C ratio is positively correlated with ba-PWV, but the participants of those studies were not limited to T2DM patients. The TG/HDL-C ratio has long been associated with insulin resistance and cardiometabolic risk. Insulin resistance states and chronic hyperinsulinemia induce the local activity of the renin-angiotensin-aldosterone system, followed by the expression of angiotensin II receptors in vascular tissue, resulting in vessel wall hypertrophy, fibrosis, and reduced arterial elasticity. Furthermore, insulin resistance is strongly associated with endothelial dysfunction, marked with an imbalance between nitric oxide (NO) and endothelin-1, two important determinants of arterial stiffness. ^8^^,^^20^

Our present study found that non-HDL-C positively correlated with arterial stiffness, and non-HDL-C levels of >150 mg/dL predict arterial stiffness with a sensitivity of 96.8% and specificity of 52.9%. An elevated non-HDL-C level also increased the risk for arterial stiffness (OR: 16.312; 95% CI: 3.760–70.780). These findings are similar to previous studies by Bando *et al*. ^21^ and de Oliveira *et al*. ^22^ which reported that elevated non-HDL-C levels are associated with an increased risk of arterial stiffness. Unlike LDL-C, the non-HDL-C represents the cholesterol content in all the atherogenic lipoproteins, namely: VLDL-C, intermediate-density lipoprotein cholesterol (IDL-C), and lipoprotein (a) cholesterol. ^23^ In T2DM patients, non-HDL-C levels emphasize the role of the TG-rich lipoproteins in the development of arterial stiffness. The TG-rich lipoproteins through the TG hydrolysis process by lipoprotein lipase (LPL) contribute to low-grade inflammation of the arterial wall. ^24^ Inflammation has a pivotal role in both arterial stiffening and atherosclerosis. ^25^ In an *in vitro* study, C-reactive protein (CRP) decreased endothelial NO synthase (NOS) secretion and activity, resulting in “functional” arterial stiffening. Furthermore, inflammation may result in dysregulation between elastin breakdown and production. Some elastolytic enzyme expressions i.e. matrix metalloproteinase-9, are induced by pro-inflammatory cytokines. ^8^

### Limitations

Our present study was limited by its cross-sectional design in nature. Other factors which might affect the association between lipid profiles or ratios and arterial stiffness, such as race-ethnicity, duration of diabetes, and medications (statins, antihypertensive, and antidiabetics), were not controlled for in this study; therefore, a bias is likely to be present.

## Conclusions

Lipid profiles and lipid ratios, especially TG/HDL-C ratio and non-HDL-C, are potential biochemical markers for arterial stiffness in T2DM patients. Type 2 DM patients with arterial stiffness were likely to have elevated lipid profiles and lipid ratios. We recommend further research with a better design (cohort) and larger scale before applying these parameters in clinical practice.

## Data Availability

Figshare: Underlying data for ‘The performance of lipid profiles and ratios as a predictor of arterial stiffness measured by brachial-ankle pulse wave velocity in type 2 diabetic patients’. https://doi.org/10.6084/m9.figshare.21572874. ^15^ Figshare: STROBE checklist for ‘The performance of lipid profiles and ratios as a predictor of arterial stiffness measured by brachial-ankle pulse wave velocity in type 2 diabetic patients’. https://doi.org/10.6084/m9.figshare.21572874. ^15^ Data are available under the terms of the Creative Commons Attribution 4.0 International license (CC-BY 4.0)
