# Supplementary material for: The performance of lipid profiles and ratios as a predictor of arterial stiffness measured by brachial-ankle pulse wave velocity in type 2 diabetic patients
Source: F1000Res. 2023 Jun 5;11:1582. Originally published 2022 Dec 23. [Version 2] doi: 10.12688/f1000research.128627.2 (PMC11364966; doi:10.12688/f1000research.128627.2)
Supplement: Peer review history

# Reviewer report: Reviewer response for version 2

**DOI:** 10.5256/f1000research.150109.r240672

**Published:** 2024-08-30

**Authors:** Alexandro J Martagón

**Affiliations:** 1 Instituto Nacional de Ciencias Médicas y Nutrición Salvador Zubirán ,The Institute for Obesity Research, Tecnologico de Monterrey, Mexico City, Mexico

Thank you for allowing me to review this interesting article.  Although I find it very complete, I suggest the following:

 The authors mention that ba-PWV is easier to perform and more convenient than cf-PWV; I would suggest to explain why is that suggested by the authors, otherwise it is not required to state so. 

 The inclusion criteria was T2DM diagnosis. Was this auto-reported from patients or it was confirmed through the recent biochemical analysis? How was the diagnosis made, fasting glucose, HbA1c, OGTT? Please specify. 

 For the ba-PWV measurement, please specify is patients were required to avoid ingesting caffeine or alcoholic beverages. If any of the patients had hypertension, specify if they were required to take the blood pressure medication, before the ba-PWV procedure.

Is the work clearly and accurately presented and does it cite the current literature?

Yes

If applicable, is the statistical analysis and its interpretation appropriate?

Yes

Are all the source data underlying the results available to ensure full reproducibility?

Yes

Is the study design appropriate and is the work technically sound?

Yes

Are the conclusions drawn adequately supported by the results?

Yes

Are sufficient details of methods and analysis provided to allow replication by others?

Yes

Reviewer Expertise:

Metabolic diseases

I confirm that I have read this submission and believe that I have an appropriate level of expertise to confirm that it is of an acceptable scientific standard.

# Reviewer report: Reviewer response for version 2

**DOI:** 10.5256/f1000research.150109.r176778

**Published:** 2024-04-25

**Authors:** Martin Haluzík

**Affiliations:** 1 Diabetes Centre, Institute for Clinical and Experimental Medicine, Prague, Czech Republic

I am happy with the current version and recommend the acceptance of the paper.

Is the work clearly and accurately presented and does it cite the current literature?

Yes

If applicable, is the statistical analysis and its interpretation appropriate?

Yes

Are all the source data underlying the results available to ensure full reproducibility?

Yes

Is the study design appropriate and is the work technically sound?

Yes

Are the conclusions drawn adequately supported by the results?

Yes

Are sufficient details of methods and analysis provided to allow replication by others?

Yes

Reviewer Expertise:

diabetes, obesity, insulin resistance

I confirm that I have read this submission and believe that I have an appropriate level of expertise to confirm that it is of an acceptable scientific standard.

# Reviewer report: Reviewer response for version 1

**DOI:** 10.5256/f1000research.141237.r174157

**Published:** 2023-05-30

**Authors:** Agata Stanek

**Affiliations:** 1 Department and Clinic of Internal Medicine, Angiiology and Physical Medicine, Medical University of Silesia, Bytom, Poland

This study aimed to explore the correlation of lipid profiles and ratios with arterial stiffness, construct a predictive model for arterial stiffness in T2DM patients, and ultimately analyze the performance (sensitivity and specificity) of the lipid ratios to detect arterial stiffness in T2DM patients. This was a cross-sectional design study conducted in the Endocrinology and Diabetes outpatient clinic at the Dr. Soetomo General Academic Hospital in Surabaya, Indonesia, during February–May 2016 and September–November 2019.

 It is quite an interesting paper. The Authors have presented sufficient data. The appropriate tables and figures have been provided. The article is easy to read and logically structured. The authors used appropriate statistical methods. The conclusions are consistent with the presented evidence and arguments.

 The Authors should address the following issues:

1. How many patients have metabolic syndrome? We know that metabolic syndrome (MS) is not a single disease but a cluster of metabolic disorders associated with increased risk for the development of diabetes mellitus and its complications. Please discuss this issue and cite the newest papers in this field.
2.  Please describe more precisely the measurement of arterial stiffness (according to which recommendations f.e.   the measurement was carried out in appropriate conditions, such as, among others: a quiet, dry room, ensuring thermal comfort; moreover, the measurement should be carried out after a ten-minute rest in a lying position. The BP value and heart rate (HR) should also be taken into account during the interpretation of the PWV value, as these parameters can influence the PWV measurement result   {doi: 10.1161/HYP.0000000000000033]

Is the work clearly and accurately presented and does it cite the current literature?

Partly

If applicable, is the statistical analysis and its interpretation appropriate?

Yes

Are all the source data underlying the results available to ensure full reproducibility?

Yes

Is the study design appropriate and is the work technically sound?

Yes

Are the conclusions drawn adequately supported by the results?

Yes

Are sufficient details of methods and analysis provided to allow replication by others?

Yes

Reviewer Expertise:

vascular medicine, PAD, thromboembolism,

I confirm that I have read this submission and believe that I have an appropriate level of expertise to confirm that it is of an acceptable scientific standard, however I have significant reservations, as outlined above.

## References

[1] : Recommendations for Improving and Standardizing Vascular Research on Arterial Stiffness: A Scientific Statement From the American Heart Association. *Hypertension* .2015;66(3) : 10.1161/HYP.0000000000000033 698-722 10.1161/HYP.0000000000000033 26160955 PMC4587661

# Response

**Published:** 2023-05-31

**Authors:** Robert Dwitama Adiwinoto

1. We appreciate the reviewer's feedback regarding the prevalence of metabolic syndrome in our research subjects. We aware that metabolic syndrome is a cluster of metabolic disorders independently associated with the development of diabetes-related complication. As a matter of fact, we have considered the components of metabolic syndrome (obesity, hypertension, and dyslipidemia) in our present study. However, we did not consider it as a condition *per se*; therefore, we described it in the table 1, using the latest criteria of metabolic syndrome as a reference in our revised paper. We also reevaluate how the presence of metabolic syndrome might affect the main findings of our study using multivariate analysis.

 2. We appreciate the reviewer's feedback regarding the measurement of arterial stiffness using the ba-PWV method. As a matter of fact, we have the ba-PWV measured under appropriate conditions, as mentioned by the reviewer. However, we did not state those conditions in the methods section; therefore, we added the procedure of ba-PWV measurement accordingly in the revised paper.

# Reviewer report: Reviewer response for version 1

**DOI:** 10.5256/f1000research.141237.r169341

**Published:** 2023-05-16

**Authors:** Martin Haluzík

**Affiliations:** 1 Diabetes Centre, Institute for Clinical and Experimental Medicine, Prague, Czech Republic

Early identification of arterial stiffness in Type 2 diabetes mellitus (T2DM) patients before the signs of overt atherosclerosis would be clinically beneficial. Current study aimed to explore the correlation of lipid profiles and ratios with arterial stiffness, and construct a predictive model for arterial stiffness in T2DM patients using those parameters. The study included one hundred and eighty-four adult T2DM patients in the diabetes outpatient clinic at the Dr. Soetomo general academic hospital in 2015 and 2019. Sociodemographic, glycosylated hemoglobin (HbA1c), lipid profiles, and brachial-ankle pulse wave velocity (ba-PWV) data were collected from all subjects. The subjects were divided into a group with arterial stiffness (ba-PWV > 18 m/sec) and without arterial stiffness (ba-PWV ≤ 18 m/sec). A correlation test was used to evaluate the association, and receiver operator characteristics (ROC) curves analysis were used to determine the cut-off value, sensitivity, and specificity. The risk analysis model was calculated using bivariate logistic regression analysis.

 The group with arterial stiffness had higher lipid profiles: total cholesterol (TC), triglyceride (TG), high-density lipoprotein cholesterol (HDL-C), low-density lipoprotein cholesterol (LDL-C), and lipid ratios. A significant positive correlation was found between TC, TG, LDL-C, and all lipid ratios with ba-PWV. A negative correlation was found between HDL-C and ba-PWV. All lipid ratio parameters can be used as predictors of arterial stiffness, especially non-HDL-C with cutoff value: 150 mg/dL (sensitivity 96.8% and specificity 52.9%) and TG/HDL-C ratio with cut-off value: 4.51 (sensitivity 81.0% and specificity 74.2%). Elevated TG/HDL-C ratio and non-HDL-C displayed higher risk (OR: 12.293 and 16.312; p < 0.05) of having arterial stiffness compared to other lipid ratios. Lipid profiles and lipid ratios, especially TG/HDL-C ratio and non-HDL-C, are potential biochemical markers for arterial stiffness in T2DM patients

 This is an interesting study on a reasonable number of patients. The paper is well written and the results are appropriate.

 I have only one minor comment: is there any reason why the authors included only lipids to their estimation. Why not include e.g. HbA1c and body weight as another important and easily obtainable parameters?

Is the work clearly and accurately presented and does it cite the current literature?

Yes

If applicable, is the statistical analysis and its interpretation appropriate?

Yes

Are all the source data underlying the results available to ensure full reproducibility?

Yes

Is the study design appropriate and is the work technically sound?

Yes

Are the conclusions drawn adequately supported by the results?

Yes

Are sufficient details of methods and analysis provided to allow replication by others?

Yes

Reviewer Expertise:

diabetes, obesity, insulin resistance

I confirm that I have read this submission and believe that I have an appropriate level of expertise to confirm that it is of an acceptable scientific standard.
